# The Impact of Chronic Limb-Threatening Ischemia on Cardiac Surgery

**DOI:** 10.3389/fsurg.2022.892309

**Published:** 2022-04-28

**Authors:** Naohiro Wakabayashi, Shinsuke Kikuchi, Naoya Kuriyama, Yuta Kikuchi, Masahiro Tsutsui, Hayato Ise, Yuri Yoshida, Daiki Uchida, Atsuhiro Koya, Tomonori Shirasaka, Nobuyoshi Azuma, Hiroyuki Kamiya

**Affiliations:** ^1^ Department of Cardiac Surgery, Asahikawa Medical University, Asahikawa, Japan; ^2^ Department of Vascular Surgery, Asahikawa Medical University, Asahikawa, Japan

**Keywords:** chronic limb-threatening ischemia (CLTI), WIfI classification, coronary artery desease, peripheral artery disease (PAD), critical limb ischaemia (CLI), cardiac surgery

## Abstract

**Purpose:**

The effect of chronic limb threatening ischemia (CLTI) on advanced cardiac disease, which requires surgical treatment, has rarely been reported. The purpose of this study was to review the outcomes of cardiac surgery in patients with CLTI and determine the risk factors, with a particular focus on the severity of CLTI.

**Patients:**

The baseline characteristics and outcomes of 33 patients who were treated for CLTI and underwent cardiac surgery were retrospectively analyzed. The states of CLTI were evaluated based on the Wound, Ischemia, and foot Infection (WIfI) classification system, and 33 patients were divided into the low-WIfI group (stages 1–2, *n* = 13) and high-WIfI group (stages 3–4, *n* = 20).

**Results:**

The in-hospital mortality rate was 0% in low-WIfI group and 35% in high-WIfI group (*p *= 0.027). Postoperative complications, particularly severe infections, occurred more frequently among high-WIfI group than low-WIfI group (70.0% vs. 23.1%, *p* < 0.01). Multivariable analysis identified foot infection grade as a WIfI classification factor and lower albumin levels as factors significantly associated with postoperative complications. The 1-year and 2-year survival rates were 84.6% and 67.7% in low-WIfI group and 45% and 28.1% in high-WIfI group, respectively (*p *= 0.011).

**Conclusions:**

Cardiac surgery in patients with high WIfI stage was an extremely high-risk procedure. In such patients, lowering the WIfI stage by lower extremity revascularization and/or debridement of diseased parts prior to cardiac surgery can be considered.

## Introduction

With aging of the population and changing lifestyles, an increasing number of patients have peripheral artery disease (PAD) worldwide (1). Chronic limb-threatening ischemia (CLTI), which is a clinical syndrome defined by the presence of PAD in combination with pain at rest, gangrene, or a lower limb ulceration >2 weeks duration, is one of the most advanced states of PAD and is associated with mortality, amputation, and impaired quality of life (2). The prognosis of patients with CLTI is poor as evidenced by the 2-year survival rate of only 60%–65% after CLTI onset; one of the most significant prognostic factors of CLTI is heart failure (3, 4). Similarly, previous studies have reported that cardiac disease, especially coronary artery disease (CAD), which is complicated with CLTI at a rate of 40%–50%, is an independent predictive factor for poor prognosis (5–8). Based on these reports, it is easy to anticipate that the prognosis of patients with CLTI requiring cardiac surgery is much poorer. In addition, it is plausible that the severity of CLTI is negatively correlated with surgical outcomes. However, there are few reports describing the relationship between CLTI and cardiac surgery. Hence, little is known about the clinical effect of CLTI on the outcomes of cardiac surgery. Moreover, although cardiac surgery risk assessment calculators, such as the Japan score, Euro score, or Society of Thoracic Surgeons (STS) score, include PAD as a risk factor, the severity of PAD is not taken into account in the calculation formulas (9–12).

The aim of this study was to review the outcomes of cardiac surgery in patients with CLTI and determine the risk factors for these pathologies, with a particular focus on the severity of PAD.

## Materials and Methods

### Study Design

Between April 2014 and August 2020, of all patients who were treated for CLTI in our institute (323 patients), 33 patients (10.2%) underwent cardiac surgery. We divided these 33 patients into two groups on the basis of the severity of CLTI according to the Wound, Ischemia, and foot Infection (WIfI) classification system (13) and retrospectively analyzed them.

Cardiac surgery was defined as only those procedures performed via median sternotomy or lateral thoracotomy approach and did not include endovascular surgeries, such as transcatheter aortic valve implantation (TAVI). Based on the state prior to cardiac surgery, the Japan score, Euro score II, and STS score were calculated as preoperative risk predictors (mortality and morbidity) using an online calculation system (9–12). The clinical data, including these risk scores, were collected from medical records with the approval of the regional ethics committee of Asahikawa Medical University (reference number: 19,209) and following the Declaration of Helsinki and the applicable ethical standards. Written informed consent was obtained preoperatively from all patients, whereby the refusal right was warranted for all patients and it was clearly documented on our homepage (http://www.asahikawa-med.ac.jp/).

### Evaluation of Limb Severity by Patient Stratification Using the WIfI Classification

The diagnosis and severity of CLTI were evaluated based on the WIfI classification system (13). This classification consists of the three major factors: “wound,” showing the severity of the foot wound; “ischemia,” assessed by the ankle–brachial index or other modalities, such as skin perfusion pressure; and “foot infection,” defined by clinical manifestations of infection. These three factors were expressed as a number from 0 to 3, in which 0 indicated no clinical manifestation and 3 indicated a serious grade. According to the WIfI score obtained based on these three factors, the WIfI clinical stage (CS), which is associated with the stratified amputation risk and has categories of 1 to 4, with 1 indicating very low risk and 4 indicating high risk, was defined. Thirty-three patients with CLTI were included as study participants and divided into two groups: the low (CS 1–2) and high (CS 3–4) WIfI classification at cardiac surgery groups.

### Therapeutic Strategy at our Institute

A therapeutic strategy was planned for each patient on the basis of the symptoms and severity of cardiac disease and CLTI, other comorbidities, and general condition, including frailty. If the cardiac condition was stable, we prioritized CLTI treatment. Cardiac surgery was prioritized in patients with unstable cardiac states, but the patients with high limb severity underwent minimal lower extremity revascularization (LER), including bypass surgery under ultrasound-guided nerve blockade or endovascular therapy (EVT), to avoid general anesthesia (14), and infectious or necrotic tissues were removed under local and/or regional anesthesia. The LER procedure, i.e., bypass surgery or EVT, was selected based on the patient’s general condition, ambulatory status, and availability of the great saphenous vein (GSV) and small saphenous vein (SSV). Throughout the treatment period, appropriate wound management was provided by the foot-care team, and appropriate antibiotics were administered on the basis of periodic bacterial culture. Therapeutic strategy and management planning were discussed among multiple disciplines and decided at the heart-vascular team conference, which also includes cardiologists in our hospital.

### Graft Selection in Patients with CAD and CLTI

As most patients with CAD and CLTI had severe systemic atherosclerosis and end-stage renal disease (ESRD), the available grafts, that is, the internal thoracic artery (ITA), radial artery (RA), gastroepiploic artery (GEA), or GSV for coronary artery bypass grafting (CABG) and the GSV, SSV, and/or upper extremity vein for LER, were often limited. The selection of grafts, especially the priority of GSV, was discussed and decided at the conference individually based on the number and severity of diseases, the availability of grafts, and the possibility of endovascular intervention.

### Endpoints

Postoperative death and adverse events during hospitalization were set as endpoints. Postoperative complications included major adverse cardiac and cerebrovascular events (MACCEs), severe infections, such as septicemia/sepsis or mediastinitis, and nonocclusive mesenteric ischemia (NOMI). Major adverse limb events (MALEs), defined as major amputation or any reintervention including acute exacerbation of CLTI and occlusion of distal bypass, were also included in the complications.

### Statistical Analysis

Categorical variables were expressed as number (n) with percentage in parentheses, and the χ^2^ test was used for comparisons. Continuous variables were expressed as mean ± standard deviation or median with interquartile range, and the Mann–Whitney *U*-test was used for comparisons. A logistic regression model was used to calculate the odds ratio with 95% confidence interval for the risk of postoperative complications. Preoperative covariable factors, including the patients’ comorbidities and limb severity, were expressed by WIfI CS. Variables exhibiting significance levels of <0.2 in the univariable analyses were assessed in the multivariable logistic regression model. *p*-values of <0.05 were considered to indicate statistical significance. All analyses were performed using SPSS version 25 (IBM Statistics for Windows, IBM Corp., Armonk, NY, USA). The survival rate after cardiac surgery was calculated using the Kaplan–Meier method, and the log-rank test was used to compare the survival rates of the two groups.

## Results

### Baseline Characteristics

The median age of all patients was 70 (41–86), and 20 (60.6%) were males. Regarding comorbidities and risk factors for arteriosclerosis, 87.9% of patients had diabetes mellitus (DM), with 41.4% being insulin users. Notably, ESRD on regular dialysis was included at a rate of 81.8%. The study participants were divided into low-WIfI group (3 patients in CS 1 and 10 patients in CS 2) and high-WIfI group (2 patients in CS 3 and 18 patients in CS 4). The baseline characteristics did not differ between the two groups (**Table 1**), except for preoperative C reactive protein levels (*p* < 0.01). The mean predicted mortality rates according to the Japan score (30-day mortality), Euro score II, and STS score (in-hospital mortality) were 13.2%, 4.53%, and 4.27% in the low-WIfI group and 10.6%, 5.37%, and 7.31% in the high-WIfI group, respectively.

**Table 1:** Comparison on baseline characteristics and cardiac/limb states between two groups.

	Overall	High WIfI group	Low WIfI group	*p* value
Variables	(*N* = 33)	(*N* = 20)	(*N* = 13)	
Patient’s characteristics				
Age, median (range)	70 (41, 86)	69.5 (41, 83)	74 (51, 86)	0.36
Male gender, *n* (%)	20 (60.6)	12 (60.0)	8 (61.5)	0.93
Diabetes mellitus, *n* (%)	29 (87.9)	18 (90.0)	11 (84.6)	1.00
Insulin use, *n* (%)	12 (36.4)	7 (35.0)	5 (38.5)	1.00
Hemodialysis, *n*	27 (81.8)	17 (85.0)	10 (76.9)	0.66
Serum albumin, Median (g/dL)	3.2 (1.8, 4.0)	3.05 (1.8, 4.0)	3.2 (2.2, 4.0])	0.31
C reaction protein, median, (range), mg/dL	0.9 (0, 7.91)	2.38 (0, 7.91)	0.31 (0, 1.91)	<0.01
Cardiac status				
LVEF, median, (range), %	55 (16, 68)	52.5 (16, 66)	58 (25, 68)	0.49
LVEF ≦ 35%, *n* (%)	10 (30.3)	7 (35.0)	3 (23.1)	0.70
Coronary artery disease (CAD), *n* (%)	26 (78.8)	16 (80.0)	10 (76.9)	1.00
Acute coronary syndromes, *n* (%)	8 (30.8)	5 (25.0)	3 (23.1)	1.00
Aortic valve stenosis, *n* (%)	15 (45.5)	8 (40.0)	7 (53.8)	0.44
Mitral valve stenosis, *n* (%)	1 (3.0)	1 (5.0)	0 (0)	1.00
Mitral valve regurgitation, *n* (%)	3 (9.1)	0 (0)	3 (23.1)	0.05
Infective endocarditis, *n* (%)	1 (3.0)	1 (5.0)	0 (0)	1.00
Endocardiac thrombus, *n* (%)	2 (6.1)	2 (10.0)	0 (0)	0.51
Aortic hard calcification, *n* (%)	8 (24.2)	6 (30.0)	2 (15.4)	0.43
Preoperative unstable hemodynamics, *n*	3 (9.1)	3 (15.0)	0 (0)	0.26
Japan score				
30-day mortality, median (range, %)	11.8 (0.9, 69.6)	10.6 (0.9, 69.6)	13.2 (2.0, 37.0)	0.52
30-day mortality + morbidity, median (range, %)	28.5 (9.5, 72.4)	30 (10.3, 72.4)	24.2 (9.5, 50.7)	0.46
Euro SCORE II				
In-hospital mortality, median (range, %)	4.86 (2.06, 33.3)	5.37 (2.06, 33.3)	4.53 (2.19, 15,2)	0.65
STS score				
In-hospital mortality, median (range, %)	6.84 (0.98, 44.5)	7.31 (0.98, 44.5)	4.27 (1.0, 27.4]	0.41
In-hospital mortality + morbidity, median (range, %)	29.0 (8.24, 81.7)	31.8 (8.24, 81.7]	25.2 (11.3, 55.0)	0.33
Limb status				
The three major factors of WIfI				
Wound grade 0/1/2/3, *n* (mean)	12/1/15/5 (1.39)	0/1/14/5 (2.20)	12/0/1/0 (0.15)	<0.01
Ischemic grade 0/1/2/3, *n* (mean)	4/2/8/19 (2.27)	1/1/3/15 (2.60)	3/1/5/4 (1.77)	<0.01
foot Infection grade 0/1/2/3, *n* (mean)	13/4/14/2 (1.15)	1/3/14/2 (1.85)	12/1/0/0 (0.08)	0.02

### Cardiac and Limb States at Cardiac Surgery

The left ventricular ejection fraction (LVEF) was fairly preserved in most cases, but 10 patients (30.3%) had reduced LVEF (≤35%). CAD (78.8%), aortic valve stenosis (AS, 45.5%), and their combination were major indications for cardiac surgery. Including the predictive risk scores, there was no significant difference in cardiac states between the two groups (**Table 1**).

Regarding limb severity, except for 2 patients suffering from resting pain, 31 (93.9%) patients had active and/or healed ischemic ulceration with or without tissue loss. Patients in the high-WIfI group had more severe grades of all factors according to the WIfI classification (**Table 1**). Despite our basic strategy, 21 (63.6%) patients had to undergo cardiac surgery with nonhealed active wounds (W 1–3) because of cardiac urgency or instability. The percentages of these patients were significantly greater in the high-WIfI group than in the low-WIfI group (100% vs. 7.7%, *p* < 0.01)

### Surgical Details and Outcomes

CABG and aortic valve replacement (AVR) were the major procedures. Emergent cardiac surgery, which is defined as surgery performed within 24 h from onset or diagnosis, was performed in eight (24.2%) patients, including those with acute coronary syndrome (ACS), left atrial thrombus, and infective endocarditis (**Table 2**). Importantly, among the 24 patients with CABG, GSV was available in only 13 (54.2%) due to previous LER (7 patients), planned LER (4 patients), poor quality, and limb wound/amputation; therefore, complete revascularization could not be achieved in these patients. Except for operative time, which was longer in the low-WIfI group, no significant difference in surgical procedures was observed between the groups.

**Table 2:** Comparison on procedures of cardiac surgery and outcomes between high WIfI group and low WIfI group.

Variables	Overall	High WIfI group	Low WIfI group	*p* value
	(*N* = 33)	(*N* = 20)	(*N* = 13)	
Surgical procedures				
Operation time, median (range), min	299 (124, 678)	287 (124, 651)	345 (239, 678)	0.047
CPB time, median (range), min	152 (62, 425)	150 (62, 425)	165.5 (75, 239)	0.56
Aortic clamp time, median (range), min	116 (46, 354)	118 (46, 354)	114 (49, 188)	0.89
Emergent operation, *n* (%)	8 (24.2)	6 (30.0)	2 (15.4)	0.43
Re-operation, *n* (%)	2 (6.1)	2 (10.0)	0 (0)	0.51
Coronary artery bypass grafting, *n* (%)	24 (72.7)	14 (70.0)	10 (76.9)	1.00
Off-pump CABG, *n* (%)	7 (21.2)	5 (25.0)	2 (15.4)	0.68
Aortic valve replacement, *n* (%)	15 (45.5)	8 (40.0)	7 (53.8)	0.44
Postoperative outcomes				
Operative death (within 30 days), *n* (%)	4 (12.1)	4 (20.0)	0 (0)	0.14
In-hospital death, *n* (%)	7 (21.2)	7 (25.0)	0 (0)	0.02
Postoperative complication^a^				
Overall, *n* (%)	17 (51.5)	14 (70.0)	3 (23.1)	0.013
Severe infections, *n* (%)	12 (36.4)	11 (55.0)	1 (9.1)	<0.01
Mediastinitis, *n* (%)	6 (18.2)	5 (25.0)	1 (9.1)	0.36
MACCE, *n* (%)	9 (27.3)	8 (40.0)	1 (9.1)	0.06
Heart failure, *n* (%)	6 (18.2)	5 (25.0)	1 (9.1)	0.36
Sudden cardiopulmonary arrest, *n* (%)	1 (3.0)	1 (5.0)	0 (0)	1.00
Cerebral infarction, *n* (%)	2 (6.1)	2 (10.0)	0 (0)	0.51
Non-occlusive mesenteric ischemia, *n* (%)	2 (6.1)	2 (10.0)	0 (0)	0.51
MALE^b^, *n* (%)	5 (15.2)	3 (15.0)	2 (18.2)	1.00

*MACCE, major adverse cardiac and cerebrovascular events; MALE, major adverse limb event.*

^a^ *Some complication occurred in the same patient.*

^b^ *MALE indicated acute exacerbation of CLTI/occlusion of distal bypass.*

The outcomes of cardiac surgery, however, were devastatingly worse in the high-WIfI group. Postoperative complications, particularly severe infections, occurred more frequently among those in the high-WIfI group (70.0% vs. 23.1%, *p* = 0.01), and seven patients in the high-WIfI group died in the hospital (**Table 2**). The 30-day mortality rate was 12.1% (4/33), which was similar to the median predicted mortality rate based on the Japan score (11.8%), whereas the in-hospital mortality rate (21.2%) was much higher than the median predicted mortality rate based on the Euro score II (4.86%) and STS score (6.84%). The postoperative complication rate (51.5%) was much higher than the median rates predicted based on the Japan score (28.5%) and STS score (29.0%).

There were 22 LERs before cardiac surgery, among which 15 (68.2%) limbs showed improvement in limb severity with reduction of the WIfI CS. Regarding the LER procedure, 32 patients (97.0%) received LER as follows: 27 patients underwent distal bypass grafting with/without EVT, whereas 5 patients underwent EVT alone. LER was not performed in the remaining patient owing to AS-induced acute heart failure and impending limb necrosis, due to which the patient died soon after cardiac surgery.

### Causes of Death and Details of Postoperative Complications

The patients in this study had concomitant complications with several fatal pathologies, such as sepsis and NOMI, and the direct cause of death and the details of major postoperative complications are listed in **Table 2**. Infection was the leading complication and cause of death both in the early and late phases after cardiac surgery, followed by cardiac events. In-hospital deaths occurred in 7 (21.2%) patients, among whom 4, 2, and 1 died due to severe infection, NOMI, and cardiac events, respectively. Moreover, death after discharge occurred in 11 (33.3%) patients, among whom 4, 3, 1, and 3 died due to severe infection, cardiac event, renal failure, and unknown causes, respectively. Severe infections were significantly more common in the high-WIfI group than in the low-WIfI group (55.0% vs. 9.1%, *p* < 0.01). All six patients who suffered from mediastinitis underwent CABG using ITA, and five of them were included in the high-WIfI group, although it did not reach statistical significance in our cohort. NOMI occurred in two patients in the high-WIfI group secondary to other complications. There was no difference in MALE occurrence between the two groups. Although the occlusion of distal bypass grafting or the exacerbation of CLTI occurred in 4 patients as a concomitant complication, the exacerbation of CLTI was observed in the wound-free limb (W0I2fI0; low-WIfI stage) of a patient without any other complication, albeit after CABG using bilateral ITAs.

Multivariable analysis revealed two significant factors associated with postoperative complications: low serum albumin levels and foot infection grade of WIfI classification (**Table 3**). When three major factors from the WIfI classification were not used in the univariable analysis, the WIfI CS was an independent factor along with serum albumin levels. This result showed that foot infection grade as a WIfI classification factor was significantly associated with postoperative complications.

**Table 3:** Risk factors of major complications during hospitalization.

Variable	Univariable analysis		Multivariable analysis	
	OR (95%CI)	*p* value	OR (95%CI)	*p* value
Age	0.99 (0.92–1.06)	0.77		
Male gender	1.4 (0.35–5.8)	0.62		
Hypertension	1.6 (0.29–8.4)	0.61		
Diabetes mellitus	3.7 (0.34–40)	0.28		
Dialysis	2.5 (0.39–16)	0.33		
Antiplatelet use	1.1 (0.13–8.7)	0.95		
Cerebrovascular disease	0.64 (0.12–3.5)	0.61		
Coronary artery disease	3.5 (0.56–21)	0.19	–	–
Severe aortic valve stenosis	0.78 (0.19–3.1)	0.72		
Ejection fraction	1.0 (0.96–1.0)	0.85		
Serum albumin	6.7 (1.3–33)	0.02	33 (1.6–100)	0.02
C-reactive protein	1.5 (0.98–1.2)	0.06	–	–
Wound grade	2.4 (1.2–5.0)	0.02	–	–
Ischemia grade	1.7 (0.82–3.6)	0.15	–	–
Foot infection grade	4.7 (1.8–12)	<0.01	9.9 (2.1–47)	<0.01
WIfI clinical stage	1.9 (1.1–3.1)	0.01	–	–
Acute coronary syndrome	3.8 (0.64–23)	0.14	–	–
Emergency operation	3.4 (0.64–23)	0.18	–	–
Aortic calcification	3.8 (0.64–23)	0.14	–	–

### Survival Rate

**Figure 1:**
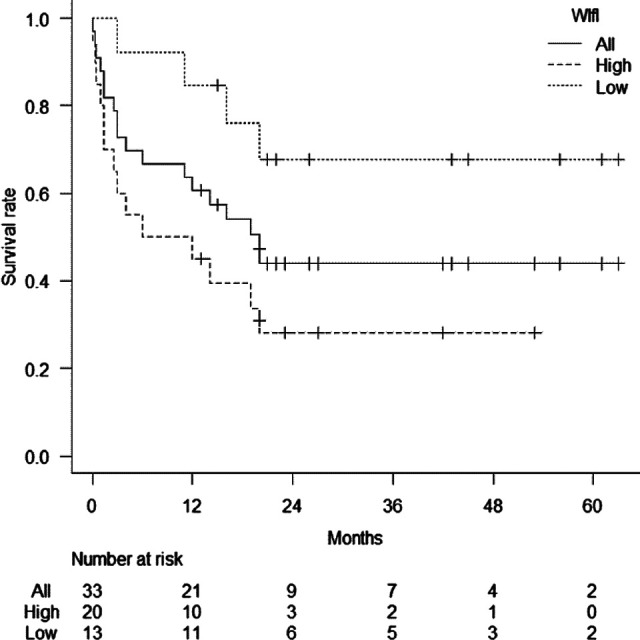
Survival rates of all patients with chronic limb-threatening ischemia after cardiac surgery. Patients were divided into two groups, high- and low-wound, ischemia, and foot infection (WIfI) clinical stage. The 3-, 6-, 12-, and 24-month survival rates were 72.7%, 66.7%, 60.6%, and 43.9%, respectively. The survival rates were significantly worse in the high-WIfI group than in the low-WIfI group.

The median follow-up period of this study was 16 months. The 3-, 6-, 12-, and 24-month survival rates were 72.7%, 66.7%, 60.6%, and 43.9%, respectively, in all patients (**Figure 1**). The survival rate was significantly lower in the patients with high-WIfI stage than in those in low-WIfI stage (*p* = 0.011); the 3-, 6-, 12-, and 24-month survival rates were 60%, 50%, 45%, and 28.1% in the high-WIfI group and 92.3%, 92.3%, 84.6%, and 67.7% in the low-WIfI group, respectively.

## Discussion

This retrospective study investigated the outcomes of cardiac surgery with comorbid active CLTI. The postoperative in-hospital mortality and morbidity rates in this study were remarkably higher than those expected based on the mean predictive risk scores. Furthermore, the midterm survival rate was remarkably poor (60.6% at 1 year and 43.9% at 2 years). In particular, the outcomes were devastating in patients with high-WIfI stage. These results were much worse than those of previous studies, which reported the 1-year survival rates of patients with PAD and CAD (15, 16), and worse than the prognosis of all patients with CLTI. This finding was undoubtedly because patients in this study had complicated advanced cardiac disease and PAD (CLTI), and to the best of our knowledge, this is the first report to describe the relationship between the outcomes of cardiac surgery and the severity of CLTI according to the WIfI classification system.

One of the most significant and debatable issues in this study was the timing of cardiac surgery. Obviously, cardiac surgery should be performed without any active wounds. The result that patients with lower WIfI stage had fewer postoperative complications and a better midterm survival rate suggests that cardiac surgery should be deferred until the CLTI wound heals as completely as possible. Although some patients required a prolonged period of foot care for wound healing, and cardiac surgery could not be deferred due to unstable cardiac conditions. For these patients, it could be important to remove the diseased sections as completely as possible before cardiac surgery based on a precise examination of the extent and depth of infections via imaging modalities, such as magnetic resonance imaging. These debriding procedures with essential LER can be performed safely with the patient under local anesthesia and nerve blockade, and this lowering or downstaging of WIfI scores may help reduce postoperative complications and improve the survival rate. In the present series, although none of the patients underwent major amputation, it may be a reasonable option for patients with high WIfI stage and extremely poor outcomes.

Nutritional status and systemic inflammation are crucial factors for perioperative management. P.L. Karas et al. reported that a low preoperative serum albumin level in patients undergoing cardiac surgery was associated with an increased risk of mortality and morbidity (17). Peacock et al. revealed a similar trend in patients undergoing LER for critical limb ischemia (18). Based on our results and these findings, patients with advanced cardiac disease and CLTI may be vulnerable to infections and coagulopathy resulting in mediastinitis, MACCE, or other complications, and perhaps easily transition to a terminal stage because of their malnourished and wasted states. Although serum albumin level is not specific to nutritional status and systemic inflammation or nephropathy were strongly associated with low serum albumin levels, optimal nutritional engagement to maintain serum albumin levels is indispensable in addition to CLTI management. Additionally, once cardiac or infectious complications occur, we must be extremely vigilant of limb states. MALE, which occurred independently from WIfI classification in this study, was also a fatal complication because in 3 out of 5 patients with MALE, the condition occurred secondary to other complications of sepsis and/or multiple organ failure, and the patients died within a few months. Concomitant MALE is thought to stem from systemic inflammation or infection, which causes hemodynamic instability requiring catecholamine use, dysfunction/injury of the endothelium, or cytokine abnormalities, causing the stagnation of blood flow and activation of the coagulation cascade (19, 20).

Faced with these severe outcomes, we believe that conventional cardiac surgery with full sternotomy is inappropriate and should be avoided, at least in patients with active CLTI (higher WIfI stage). Full sternotomy might be acceptable in cases of cardiac emergency, but a less-invasive intervention should be considered as a bailout procedure. Several studies regarding the strategy against multivessel CAD have reported that compared with conventional off-pump CABG, hybrid revascularization (minimally invasive cardiac surgery (MICS)-CABG combined with percutaneous coronary intervention) provided shorter postoperative recovery and similar excellent short- and long-term outcomes (21, 22). Moreover, MICS-AVR or TAVI should help reduce postoperative complications, particularly mediastinitis, although access to the procedure is limited and the long-term outcomes of TAVI for patients with hemodialysis are unknown.

This study had some limitations. First, this was a retrospective single-center study with a small sample size. Second, although the treatments against cardiac disease and CLTI were conducted according to our basic therapeutic strategy, the actual clinical settings were more complicated. Most patients in this study had DM and concomitant neuropathy, only a few patients had minor symptoms, including patients with ACS. In addition, revascularization strategy for CAD and CLTI depends on an individual condition. Especially, high-WIfI group was likely to prioritize CLTI treatment due to limb severity. Therefore, the timing of cardiac surgery was indefinite and not always formalized. Third, the adverse effects of prioritized LER on cardiac stability or postoperative course after cardiac surgery could not be determined in the study. Thus, further prospective studies are warranted to investigate this.

## Conclusion

Cardiac surgery in patients with advanced CLTI and high-WIfI stage was found to be an extremely high-risk procedure. In such patients, lowering the WIfI stage by LER and/or debridement, including major/minor amputation of diseased parts, prior to cardiac surgery should be considered.

## Data Availability

The raw data supporting the conclusions of this article will be made available by the authors, without undue reservation.
